# Differential escape of neutralizing antibodies by SARS-CoV-2 Omicron and pre-emergent sarbecoviruses

**DOI:** 10.21203/rs.3.rs-1362541/v1

**Published:** 2022-02-23

**Authors:** Lin-Fa Wang, Chee Wah Tan, Wan Ni Chia, Feng Zhu, Barnaby Young, Napaporn Chantasrisawad, Shi-Hsia Hwa, Aileen Ying-Yan Yeoh, Beng Lee Lim, Wee Chee Yap, Surinder Kaur Pada, Seow Yen Tan, Watsamon Jantarabenjakul, Shiwei Chen, Jinyan Zhang, Yun Yan Mah, Vivian Chen, Mark Chen, Supaporn Wacharapluesadee, COMMIT-KZN Team, Opass Putcharoen, David Lye

**Affiliations:** Duke-NUS Medical School; Duke-NUS Medical School; Duke NUS Graduate Medical School; Duke-NUS Medical School; National Centre for Infectious Diseases; Thai Red Cross Emerging Infectious Diseases Clinical Center, King Chulalongkorn Memorial Hospital,; Africa Health Research Institute; Duke-NUS Medical School; Duke-NUS Medical School; Duke-NUS Medical School; Ng Teng Fong General Hospital; Changi General Hospital; Chulalongkorn University; Duke-NUS Medical School; Duke-NUS Medical School; Duke-NUS Medical School; Duke-Nus Medical School; NCID Singapore; Thai Red Cross EID Health Science Centre; Africa Health Research Institute; King Chulalongkorn Memorial Hospital; National Centre for Infectious Diseases

## Abstract

The SARS-CoV-2 B.1.1.529 lineage, Omicron variant, was first detected in November 2021 and carries 32 amino acid mutations in the spike protein (15 in RBD) and exhibits significant escape of neutralizing antibodies targeting the parental SARS-CoV-2 virus. Here, we performed a high-resolution multiplex (16-plex) surrogate virus neutralization assay covering all major SARS-CoV-2 variants and pre-emergent ACE2-binding sarbecoviruses against 20 different human serum panels from infected, vaccinated and hybrid immune individuals which had vaccine-breakthrough infections or infection followed by vaccination. Among all sarbecoviruses tested, we observed 1.1 to 4.7-, 2.3 to 10.3- and 0.7 to 33.3-fold reduction in neutralization activities to SARS-CoV-2 Beta, Omicron and SARS-CoV-1, respectively. Among the SARS-CoV-2 related sarbecoviruses, it is found that the genetically more distant bat RaTG13 and pangolin GX-P5L sarbecoviruses had less neutralization escape than Omicron. Our data suggest that the SARS-CoV-2 variants emerged from the changed immune landscape of human populations are more potent in escaping neutralizing antibodies, from infection or vaccination, than pre-emergent sarbecoviruses naturally evolved in animal populations with no or less immune selection pressure.

## Introduction

**Figure 1:**
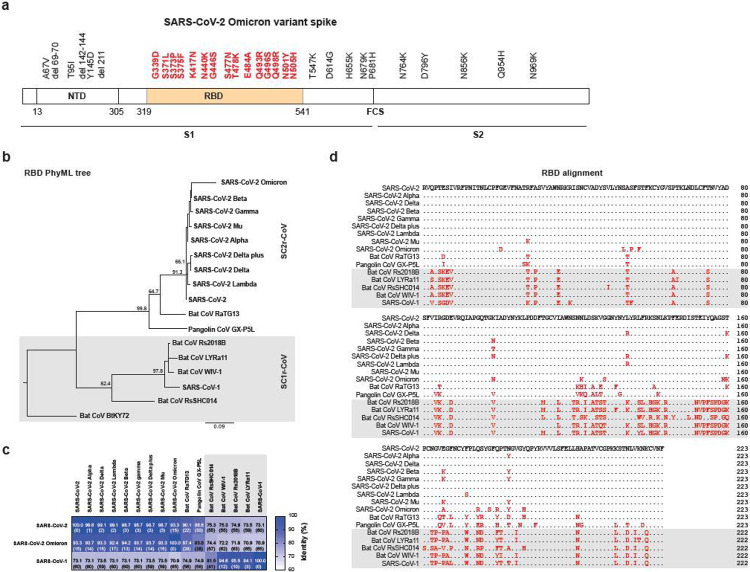
Mutations in Omicron Spike protein and RBDs of SARS-CoV-2 variants and other sarbecoviruses. **a,** Illustration of SARS-CoV-2 Omicron spike mutations. Mutations in the RBD region are highlighted in red. **b,** Phylogenetic tree based on the amino acid sequence of RBD was generated using PhyML with Blosum62 model with 1,000 bootstrap replicates. Numbers at the branches are percentage bootstrap values for the associated nodes. Scale bar indicates number of substitutions per site. **c,** RBD amino acid sequence identity (%) among different sarbecoviruses. Numbers in brackets indicates the total number of amino acid residue differences from the ancestral SARS-CoV-2 RBD. **d,** Multiple alignment of amino acid sequences of sarbecovirus RBDs used in this study. Mutations/deletions were highlighted in red.

The coronavirus disease 2019 (COVID-19) pandemic started in December 2019 and has caused 364 million cases and claimed 5.6 million lives, as of 27 January 2020. The causative agent, SARS-CoV-2^1^, is a member of the subgenus *Sarbecovirus*, same as for the SARS-CoV-1 that caused the SARS outbreak 19 years ago^2^. Bats are known reservoirs for SARS-related coronaviruses^3,4^. Multiple sarbecoviruses have been detected in bats^5-8^ and more recently in pangolins^9^. SARS-CoV-2 variants of concern (VOC) emerged since late 2020 after 11 months of evolution via massive human-to-human transmission, probably in response to the changing immune dynamics of the human population^10^, with five major VOCs recognized by WHO hitherto, namely Alpha, Beta, Gamma, Delta and Omicron. SARS-CoV-2 VOCs have either developed resistance/escape to neutralizing antibodies^11-16^ or acquired mutations that enhanced transmission or pathogenicity^17,18^. The SARS-CoV-2 Omicron variant was first detected in South Africa and Botswana and has rapidly spread to many countries as the most rapidly transmitting VOC to date. There are two major features making Omicron different from previous VOCs: it contains the most mutations in the spike protein (32 aa mutations) and the receptor binding domain (15 aa mutations) (Fig. 1a) and it is much more capable of escaping neutralizing antibodies (NAbs) than any prior VOC^19-22^ (Fig 1a). The enhanced transmission phenotype of Omicron is mostly linked to its ability of NAb escaping.

There have been several published studies comparing effectiveness of NAbs on Omicron versus the parental strain or other VOCs using conventional live virus neutralization test (cVNT) or spike pseudovirus VNT (pVNT) using either infected or vaccinated serum panels. Due to the inter-assay variability and labor-intensive nature of cVNT and pVNT, it is difficult, if not impossible, to conduct a pan-sarbecovirus NAb-escape study using a large number of human immune sera. Our current study aims to fill this gap. Here we employed a 16-plex surrogate virus neutralization test (sVNT) platform to provide a high-resolution examination of NAb-escape characteristics of all known human SARS-CoV-2 VOCs as well as pre-emergent sarbecoviruses of bat and pangolin origin. The sVNT developed in early 2020^23,24^ has been validated and applied in multiple studies examining NAbs from infected, vaccinated and vaccine-breakthrough cohorts as well as virus origin and human-to-animal transmission studies^5,25-28^. The sVNT measures RBD-targeting NAbs, which accounts for more than 90% of total NAbs^29,30^.

Combining this powerful NAb detection platform with a large collection of 20 different human serum panels, we found that Omicron’s ability to escape NAbs is more potent than one would expect from a natural virus evolution path in a non-infected or unvaccinated population, pointing to the possibility of Omicron emergence as a result of immune selection during human transmission in a changed immune landscape after almost two years of SARS-CoV-2 circulation and vaccination program around the globe.

## Results

### High resolution assessment of RBD-targeting sarbecovirus neutralizing antibodies by a 16-plex surrogate virus neutralization test (sVNT) platform

With the increasing number of SARS-CoV-2 VOCs and pre-emergent animal sarbecoviruses discovered in recent times, we have extended our original sVNT platform^23,24^ to a 16-plex assay which includes 11 RBD proteins of the SARS-CoV-2 clade (clade-2) viruses and 5 RBDs of the SARS-CoV-1 clade (clade-1) viruses (Fig. 1). Clade-2 viruses include the ancestral SARS-CoV-2 virus (Wuhan-hu-1), variants of concern or interest (Alpha, Delta, Beta, Gamma, Delta plus, Lambda, Mu, and Omicron) and pre-emergent bat (RaTG13) and pangolin (GX-P5L) sarbecoviruses. For clade-1, we included human SARS-CoV-1 and pre-emergent bat viruses (Rs2018B, LYRa11, RsSHC014 and WIV1) (Fig 1b-d, Supplementary Data Fig 1). In comparison to the SARS-CoV-2 ancestral RBD, the other 15 RBDs have the following rank of sequence relatedness (from high to low): Alpha, Delta, Lambda, Beta, Gamma, Delta plus, Mu, Omicron, RaTG13, GX-P5L, RsSHC014, WIV-1, Rs2018B, LYRa11 and SARS-CoV-1. It is interesting to note that the reverse order in comparison to SARS-CoV-1 RBD is not exactly identical (Fig. 1b, Supplementary Data Fig 1). For example, Omicron is the most distantly related to SARS-CoV-1, containing 65 aa mutations in the RBD region.

As expected, all RBDs bind to human ACE2 in a dose-dependent manner in the multiplex Luminex system. The Omicron RBD showed the weakest binding in this assay system (Extended Data Fig. 1a). We first tested the 16-plex sVNT using the WHO international standard 20/136^31^ and the neutralization activities were reduced from SARS-CoV-2 in the following descending order for the human SARS-CoV-2 variants: Delta, Alpha, Delta plus, Lambda, Gamma, Beta, Mu and Omicron. Only limited cross-reactivity to SARS-CoV-1 and clade-1 sarbecoviruses was observed (Extended Data Fig 1b). The calibration of WHO international standard using SARS-CoV-2 ancestral multiplex sVNT was modelled (Extended Data Fig 1c) and the IU/ml at 1:80 dilution of all the tested serum panels in this study are listed in Supplementary Data Table 1. To ensure that our RBD-targeted sVNT assay is well correlated with NAb assays based on whole spike protein, we conducted a comparative analysis using the multiplex sVNT and pseudovirus-based VNT (pVNT) against the ancestral and Omicron viruses, respectively. From a well-defined panel of 120 sera with varying levels of neutralizing antibodies (NAbs), the data showed a good correlation between sVNT and pVNT with R^2^ of 0.83 and 0.73 for the ancestral and Omicron viruses, respectively (Extended Data Fig 1d, 1e).

### Reduction of neutralization activities of different human serum panels to SARS-CoV-2 Omicron

After confirming the functional performance of the 16-plex sVNT platform, we examined the ability of different human serum panels to neutralize Omicron. The serum panels were in three different groups: 1) convalescent sera from natural infection (4 panels), 2) serum from vaccinees, with or without the booster (12 panels); and 3) the hybrid immune group which had a mixture of infection and vaccination or vice versa (4 panels). See Supplementary Data Table 2 for details of the 20 different serum panels used in this study.

**Figure 2:**
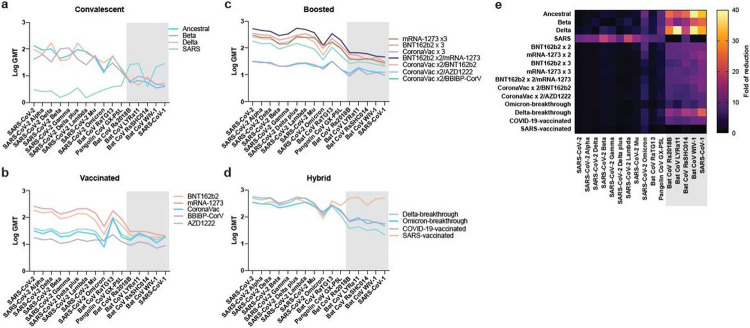
Neutralization activity against SARS-CoV-2 variants and other sarbecoviruses. The geometric mean neutralization titer 50% (GMT) against 16 different sarbecoviruses derived from four different serum panels. **a,** Convalescent sera from individuals infected with Ancestral, VOCs Beta, Delta or SARS-CoV-1; **b,** Individuals who have received standard vaccination (BNT162b2 x2, mRNA-1273 x 2, CoronaVac x2, BBIBP-CorV x2 and AZD1222 x2); **c,** Individuals after receiving booster (BNT162b2 x3, mRNA-1273 x3, BNT162b2 x2/mRNA-1273, CoronaVac x3, CoronaVac x2/BNT162b2, CoronaVac x2/AZD1222, and CoronaVac x2/BBIBP-CorV); and **d**, Individuals with hybrid immunity (Delta-breakthrough, Omicron-breakthrough, COVID-19-vaccinated, and SARS-vaccinated). **e**, GMT fold reduction heat-map of different convalescent, vaccine and hybrid immunity panels against 16 different sarbecoviruses.

For group 1: we had access to convalescent sera from patients infected with SARS-CoV-2 ancestral, Beta and Delta variants and SARS-CoV-1. Sera from all three SARS-CoV-2 related viruses had reduced level of neutralization against Omicron, with more significant reduction from the Ancestral- and Beta-infected patients, with geometric mean NT50 of 10.3-fold and 6.7-fold reduction from the homologous RBD, respectively (Fig 2a, e, Supplementary data Fig 2a).

For group 2: we employed a total of 12 different serum panels including homologous and heterologous boosted individuals. Those who received two doses of BNT162b2 and mRNA-1273 have significant reduction of NAbs to Omicron, with 8.1 and 5.7-fold reduction, respectively. The level of NAbs to all viruses induced by two doses of inactivated vaccine and viral vector-based vaccine are lower than the mRNA vaccines (Fig 2b, Supplementary Data Fig 2b). Vaccinated individuals who received a third dose of BNT162b2, mRNA-1273 or AZD1222, but not inactivated vaccines, significantly increased overall NAb titer to SARS-CoV-2 and variants (Fig 2c, Supplementary Data Fig 2c). But the NAb titer to Omicron is significantly lower than ancestral, with 3.7, 4.2, 4.9 and 5.0-fold reduction for BNT162b2 x 3, BNT162b2 x2/mRNA-1273, CoronaVac x 2/BNT162b2 and CoronaVac x 2/AZD1222, respectively (Fig 2c and 2e, Supplementary Data Fig 2c).

For group 3: despite the reported enhancement of level and breadth of hybrid immunity in the infection-vaccination or vaccination-infection groups, significant reduction of NAb level to Omicron was observed in our study, revealing 2.3, 4.4, 4.5 and 3.9-fold reduction for the Omicron-breakthrough, Delta-breakthrough, COVID-19-vaccinated and SARS-vaccinated groups, respectively (Fig 2d and e, Supplementary Data Fig 2d). It is interesting to note that the NAb level to Omicron is similar between the Omicron- and Delta-breakthrough groups. None of the vaccine-breakthrough and COVID-19-vaccinated individuals had neutralization activity to clade-1 sarbecoviruses. In other words, the SARS-vaccinated group is the only one who had genuine pan-sarbecovirus NAbs despite the fact that they also suffered some level of reduction in neutralization against Omicron.

### Correlation between degree of neutralizing antibody escape and number of RBD mutations

**Figure 3:**
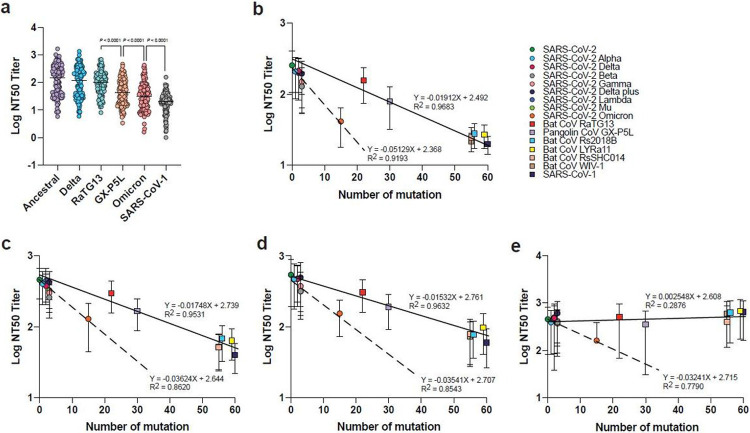
Neutralization escape in relation to number of RBD mutations. **a,** Neutralization titers derived from multiplex sVNT (n = 124). The effect of RBD mutations on NAb escape for different serum panels including those with **b,** two doses of mRNA vaccines, **c,** three doses of mRNA vaccine, **d,** vaccinated individuals with breakthrough infection, and **e,** BTN162b2-vaccinated SARS survivors. Paired two-tailed student’s t-tests were used in **a**. Line in **a** indicates median. Linear regression analysis in **b-e** were performed using GraphPad prism. Line and dotted line in **b-e** indicates linear regression plot on SARS-CoV-2 with pre-emergent sarbecoviruses and VOCs, respectively.

Bat CoV RaTG13 and pangolin CoV GX-P5L are both phylogenetically placed in the SARS-CoV-2 clade, which are more distantly related to SARS-CoV-2 than Omicron with 90.1% and 86.6% amino acid sequence identity, respectively, of the RBD sequence in comparison to Omicron at 93.3% (Fig 1b, c). When the level of NAb titers were examined side by side, it was found that Omicron had a more significant NAb escape than either RaTG13 or GX-P5L (Fig 2a-d, Fig 3a, Supplementary Data Fig 1a-d), indicating that Omicron is antigenically more distant than the two pre-emergent sarbecoviruses despite the fact that there are 15 aa differences in Omicron RBD in comparison to 22 and 30 aa differences in the RBD of RaTG13 and GX-P5L, respectively. Our data further indicated that the degree of NAb escape in VOCs was more significant than that exhibited by pre-emergent sarbecoviruses of animal origin using serum panels from 2-dose mRNA vaccinated (Fig 3b), 3-dose mRNA vaccinated (Fig 3c) and vaccine-breakthrough (Fig 3d) groups. In other words, to achieve the same level of NAb escape, the number of aa mutations in the RBD region needs to be greater than those in the VOCs. Importantly, it was shown that the degree of NAb escape was less for the BNT162b2-vaccinated SARS survivor group with the exception of VOC Omicron (Fig 3f), again highlighting the greater-than-expected NAb escape displayed by Omicron in relation to the number of aa mutations in the RBD sequence.

Apart from phylogenic distance based on genetic differences, antigenic cartography was used to generate antigenic maps that could reflect the antigenic properties of closely related pathogens^32,33^. Generated from neutralization titer 50% of the examined 187 sera on all 16 sarbecoviruses (Supplementary Data Table 1), the antigenic map showed the clustering of SARS-CoV-2 clade and the antigenic distance between the SARS-CoV-2 and other clade 2 sarbecoviruses in the following descending order: Delta plus, Delta, Lambda, Alpha, Gamma, Mu, RaTG13, Beta, GX-P5L and Omicron (Extended Data Fig 2a). Among all clade-2 sarbecoviruses, only Omicron showed an extremely large distance (>2 Antigenic Unit) between SARS-CoV-2, revealing the unusual antigenic drift of Omicron. Similar observation is observed with vaccinated serum panels (Extended Data Fig 2b). On the antigenic map generated from SARS-vaccinated serum panel (Extended Data Fig 2c), the antigenic distance between SARS-CoV-2 clade and SARS-CoV-1 clade coronaviruses was closer, yet the Omicron is antigenic distinct. Consistent with our earlier observation, Omicron is more antigenic distant to SARS-CoV-2 than GX-P5L or RaTG13.

To further dissect the differential level of NAb escape in relation to RBD aa mutations, we employed a panel of hyperimmune rabbit sera raised against recombinant RBD proteins of different clade-2 sarbecoviruses, including human SARS-CoV-2, bat CoV RaTG13, pangolin CoV GX-P5L, and two non-ACE2 binding RBDs from bat CoV RmYN02 and bat CoV SL-ZC45^5^. Consistent with the previous data obtained from human sera, the SARS-CoV-2 RBD rabbit hyperimmune sera exhibited a 9.1-fold reduction of NAb titers against Omicron. Sera raised against RaTG13 and GX-P5L RBDs had highest NAb titer to their homologous viruses in the 16-plex sVNT and displayed a 6.8-fold and 4.1-fold NAb titer reduction, respectively, to SARS-CoV-2 (Extended Data Fig 3a, b). In contrast, Omicron showed an almost complete NAb escape against these sera, with a 64.8-fold and 33.2-fold reduction, respectively, against the RaTG13 and GX-P5L hyper immune sera (Extended Data Fig 3a, b), again demonstrating the greater NAb escape ability unique to Omicron in the context of RBD mutations or sequence relatedness. Sera derived from SARS-CoV-1 immunized rabbits exhibited 28.4-fold reduction to Omicron. For the hyperimmune sera raised against the non-ACE2 binding RBDs of RmYN02 and SL-ZC45, it was interesting to note that they had the highest NAb titers against VOC Delta Plus, with GMT of 86 and 289, respectively (Extended Data Fig 3c). As a negative control, no NAb was detected for the rabbit hyperimmune sera raised against a non-sarbecovirus bat CoV HKU1 RBD (Extended Data Fig 3c).

## Discussion

In less than a year after COVID-19 pandemic, vaccine candidates based on the original Wuhan-hu-1 (ancestral) strain spike protein were developed, including mRNA, inactivated, protein subunit and virus-vectored vaccines. As of 27 January 2022, more than 9.8 billion doses of vaccine have been administered worldwide. Of the vaccine induced immunity, NAb levels are highly predictive of immune protection from symptomatic SARS-CoV-2 infection^34^. With the great ability of Omicron in NAb escape, it has been shown that an mRNA-based vaccine booster is essential to increase NAb to protect against Omicron infection^19-22,35^ and our data in this study support previous findings (Fig 2c, e). Our study further demonstrates that three doses of inactivated virus vaccine failed to elicit sufficient NAbs against Omicron (Fig 2c). However, for those who have received two doses of inactivated virus vaccines, heterologous boosting with either mRNA vaccine BNT162b2 or viral-vectored vaccine AZD1222 could induce significantly higher amount of NAbs against Omicron (Fig 2c). Breakthrough infection significantly increased overall NAb titers against all SARS-CoV-2 variants, including Omicron (Fig 2d). In general, Delta-breakthrough infection seems to elicit higher levels of NAbs against different clade-2 sarbecoviruses than Omicron-breakthrough infection (Fig 2d), probably due to the less pathogenic nature of the Omicron virus ^36,37^. Despite showing reduced NAb activities against Omicron, BNT162b2-vaccinated SARS survivors remains the only group demonstrating true pan-sarbecovirus NAbs^23^ (Fig 2d-e).

It should be noted that our sVNT is based on detection of RBD-targeted NAbs by blocking RBD-ACE2 interaction as a biochemical simulation of a genuine live virus-based VNT. -For that reason, the sVNT will be less sensitive as it is unable to detect the total NAbs against the whole Spike protein as in PRNT or pVNT assays. -The fold of reduction in our study is generally less than those published in previous studies using pVNT or PRNT. -However, the comparative fold of reduction across different sarbecoviruses or different serum panels is consistent regardless of the VNT platform used.

Bats are known natural reservoir for SARS-related coronaviruses^3,4^. Rich diversity of SARS-related coronaviruses detected in bats and pangolins highlight the risk of future spillover event^8,9,38,39^. The emergence of viral variants is within expectation with massive transmission events happening since the start of pandemic. Drivers for variant emergence include change of population immunity landscape^10^, selective pressure from convalescent plasma or monoclonal antibody therapy on immunocompromised or chronically infected individuals^40-42^ and reverse zoonosis^25,43,44^. The findings presented in this study would suggest that the emergence of Omicron is most likely due to host’s immune selective pressure^45^. This is based on the fact that pre-emergent clade-2 sarbecovirus GX-P5L and RaTG13, which are evolutionarily more distantly related to SARS-CoV-2 than Omicron (Fig 1b), showed less degree of NAb escape than Omicron (Fig 3a-e). This is true in general for VOCs in comparison to pre-emergent sarbecoviruses of animal origin which are unlikely to be exposed to SARS-CoV-2 related immune selective pressure.

Our high-resolution sVNT analysis indicated the presence of four major antigenic clades based on cross-NAb activities: SARS-CoV-2 and clade-2 sarbecoviruses (minus Omicron), VOC Omicron, bat CoV RsSHC014, and SARS-CoV-1 and its related bat viruses. It is important to note that bat RaTG13 and pangolin GX-P5L, but not Omicron, are antigenically grouped with SARS-CoV-2. This was first shown from the human sera studies, subsequently supported by the cross-NAb activities of the rabbit hyperimmune sera raised against the RBDs of these two viruses, i.e., these rabbit sera neutralized SARS-CoV-2 and most variants of concern, but not Omicron (Extended Data Fig 3a-c).

In conclusion, our study demonstrated the following important observations and findings. First, a high resolution NAb assessment platform is highly powerful in dissecting differential NAb escape abilities in the context of its correlation with RBD mutations. Second, the large number of different human serum panels provided extra confidence in confirming the greater-than-expected NAb escape ability of Omicron. Third, it is clear that Omicron is exceptional in its NAb escape capability when considering that GX-P5L, with twice as many RBD mutations, displayed less degree of NAb escape, suggesting that the emergence of Omicron is likely due to immune selective pressure. Fourth, Omicron breakthrough infections did not induce higher or broader NAbs against clade-2 sarbecoviruses in comparison to Delta breakthrough infections. Fifth, among all the human serum panels, the BNT162b2-vaccinated SARS survivors is the only group that displayed true pan-sarbecovirus NAbs.

The emergence of Omicron highlights the urgent needs to develop more broadly protective vaccines which can act in a VOC-agnostic manner. Despite 15 aa mutations in its RBD, Omicron already shows a distinctive antigenic grouping different from all other clade-2 sarbecoviruses. It is highly possible that such variants may emerge if VOC-specific vaccines are mass deployed as we have experienced with the two major VOCs, Delta and Omicron, in 2021 since mass vaccination started for the ancestral viral strain. Data from the Omicron breakthrough infection group further suggest that an Omicron-specific vaccine is unlikely to provide the pan-sarbecovirus NAbs required to prevent future VOC infection. Data from the vaccinated SARS survivors would argue that cross-clade immunization approach may be the most effective in inducing genuinely pan-sarbecovirus NAbs.

## Methods

### Human serum panels

Naïve control sera (n = 10) were used as control. Convalescence plasma/sera with primary parental infection were SARS-CoV-2 (n = 10), beta variant (n = 10), delta variant (n = 10) and SARS-CoV-1 (n = 10). Individuals who received two doses of COVID-19 vaccine, at 14 days post second dose, comprised of two doses of BNT162b2 (n = 10), mRNA-1273 (n = 10), CoronaVac (n = 10), BBIBP-CorV (n = 10) and AZD1222 (n = 10). Individuals who received booster shots after two doses of COVID-19 vaccines comprised of homologous booster (BNT162b2 x 3 n = 8; mRNA-1273 x 3, n = 7; CoronaVac x 3, n = 10) and heterologous booster (BNT162b2 x2/mRNA-1273, n = 6; CoronaVac x2/BNT162b2; CoronaVac x2/AZD1222, n = 10). Delta-breakthrough infection after two doses of mRNA vaccine recipients, n = 16; Omicron-breakthrough infection after two doses of mRNA vaccine recipients, n = 7. Individuals with prior exposure to SARS (n = 9) and COVID-19 (n = 10) followed by one dose of BNT162b2 mRNA vaccine. Ethics and IRB approvals are listed in Supplementary Data Table 2.

### Rabbit hyperimmune sera raised against recombinant RBD proteins

Rabbit anti-SARS-CoV-2, GX-P5L, RaTG13, RmYN02, SL-ZC45 and HKU1 RBD sera were all custom produced by Genscript. Rabbit anti-SARS-CoV-1 sera were described in previous studies^46^.

### Cell line

Lung carcinoma epithelial (A549, ATCC CRM CCL-185) cells were grown and maintained in RPMI-1640 medium supplemented with 10% fetal bovine serum (FBS). A549-ACE2 cells were produced by transduction of 3^rd^ generation lentiviruses carrying human ACE2 gene under EF1-alpha promoter in pFUGW vector. A549-ACE2 cells were maintained in RPMI-1640 supplemented with 10% FBS and 15 μg/ml of blasticidin.

### Sarbecovirus RBDs for the 16-plex sVNT assay system

The RBDs included in this study are as follows. A) Clade-2 sarbecoviruses: SARS-CoV-2 Ancestral, SARS-CoV-2 VOCs (Alpha, Beta, Gamma, Delta, Omicron), SARS-CoV-2 variants of interest (Delta plus, Lambda, Mu), bat CoV RaTG13, pangolin CoV GX-P5L); B) Clade-1 sarbecoviruses: SARS-CoV-1 and bat CoVs WIV-1, Rs2018B, LYRa11 and RsSHC014.

### Enzymatic biotinylation of recombinant RBD proteins

Biotinylated RBD proteins from ancestral SARS-CoV-2, SARS-CoV-2 Alpha, Delta, Beta, Gamma, bat CoV RaTG13, Pangolin CoV GX-P5L and SARS-CoV-1 were custom-made by Genscript. Biotinylated SARS-CoV-2 Omicron RBD was purchased from Acrobiosystems. Biotinylated RBDs from SARS-CoV-2 Delta plus, Mu and Lambda, bat CoVs WIV1, Rs2018B, LYRa11 and RsSHC014 were produced in-house. Briefly, the RBD coding sequences were cloned into pcDNA3.1 vector with SARS-CoV-2 signal peptide (amino acid 1-14) at the N-terminus and 10x his-tag followed by AviTag at the C-terminus. After transfection of expression plasmid into HEK293T cells using FuGENE6 in Opti-MEM media, expressed proteins were harvested at day 3 or day 6 post-transfection, respectively. RBD proteins were purified using Ni Sepharose (GE Healthcare) and desalted using Amicon Ultra-4, 10K MW (Merck). Enzymatic biotinylation of AviTag was performed using BirA Protein-Biotin ligase kit (Avidity) according to the manufacturer’s instructions. Excessive biotin was removed by Amicon Ultra-4, 10 MW (Merck). Protein concentration was determined by Nanodrop (DeNovix).

### Multiplex surrogate virus neutralization test

Multiplex sVNT were established as previously described^23^. Briefly, AviTag-biotinylated RBD proteins was coated on MagPlex-Avidin microspheres (Luminex) at 5 μg per 1 million beads. RBD-coated beads (600 per antigen) were pre-incubated with testing serum at final dilution of 1:20, 1:80, 1:320, 1:1280) for 15 min at 37°C with agitation, followed by addition of 50 μl of PE conjugated human ACE2 (2 mg/ml; Genscript) and incubated for an additional 15 minutes at 37°C with agitation. After two washes with 1 % BSA in PBS, the final readings were acquired using the MAGPIX system (Luminex) following manufacturer’s instruction.

### Pseudovirus neutralization test (pVNT)

SARS-CoV-2 Wuhan-hu-1 (ancestral), delta, Omicron and GX-P5L full-length spike pseudotyped viruses were produced and packaged as previously described^23,24^. Briefly, 5 million of HEK293T cells were transfected with 20 μg of pCAGGS spike plasmid using FuGENE6 (Promega). At 24h post transfection, cells were incubated with VSVΔG luc seed virus (at MOI of 5) for 2h. Following two PBS washes, infected cells were replenished with complete growth media supplemented with 1:5000 diluted anti-VSV-G mAb (Clone 8GF11, Kerafast). At 24h post infection, pseudoviruses were harvested by centrifugation at 2,000 x g for 5 min. For pVNT assay, 3 x 10^6^ RLU of pseudoviruses were pre-incubated with four-fold serial diluted test serum in a final volume of 50 μl for 1h at 37°C, followed by infection of ACE2-stablely-expressing A549 cells. At 20-24h post-infection, an equal volume of ONE-Glo luciferase substrate (Promega) was added and the luminescence signal was measured using the Cytation 5 microplate reader (BioTek) with Gen5 software version 3.10.

### Phylogenetic analysis

ACE2-binding sarbecovirus RBD sequences were either directly retrieved from NCBI or translated from nucleotide sequences retrieved from GISAID. Further analysis was performed in the Geneious Prime (version 2021.1). RBD protein sequences were aligned with MAFFT to construct the phylogenetic tree by the maximum-likelihood method with the blosum62 model using 1,000 bootstrap replicates in the PHYML 3.0 software.

### Antigenic cartography

Antigenic map was generated using the R package “Racmacs” (version 1.1.18) in R (version 4.1.2) based on the matrix of neutralization titer 50% of serum on the 16 sarbecoviruses on multiplex sVNT. The number of optimizations was set to 1000.

### Statistical analysis

Statistical analysis was performed using GraphPad Prism 8 software. The differences between groups were analysed using unpaired t-test. Correlations between sVNT and pVNT were analysed using Pearson correlation coefficients.

## Supplementary Material

Supplement 1

Supplement 2

Supplement 3

Supplement 4
