# Supplementary figures and images for: Differential escape of neutralizing antibodies by SARS-CoV-2 Omicron and pre-emergent sarbecoviruses
Source: Res Sq. 2022 Feb 23:rs.3.rs-1362541. Preprint. [Version 1] doi: 10.21203/rs.3.rs-1362541/v1 (PMC8887082; doi:10.21203/rs.3.rs-1362541/v1)

**a**

ACE2 binding RBD

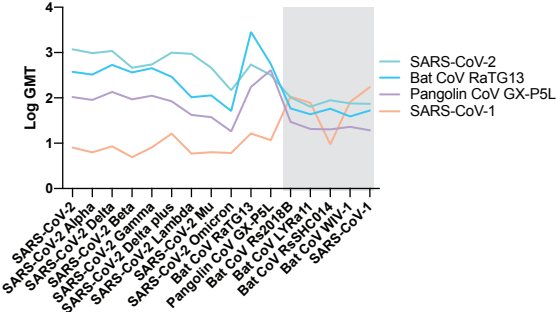

**b**

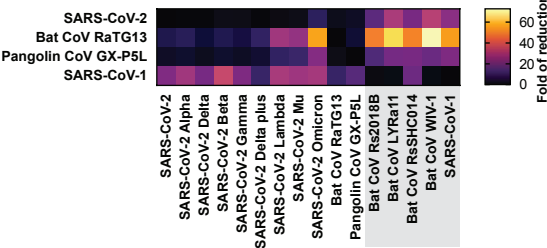

**c**

non-ACE2 binding RBD

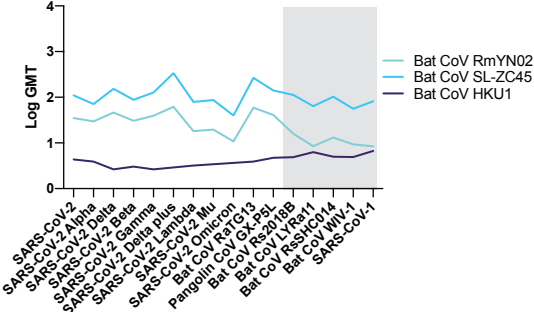

Supplement: Supplement 1 [file 5ba4e1e086f4c1f74e962d65.pdf]

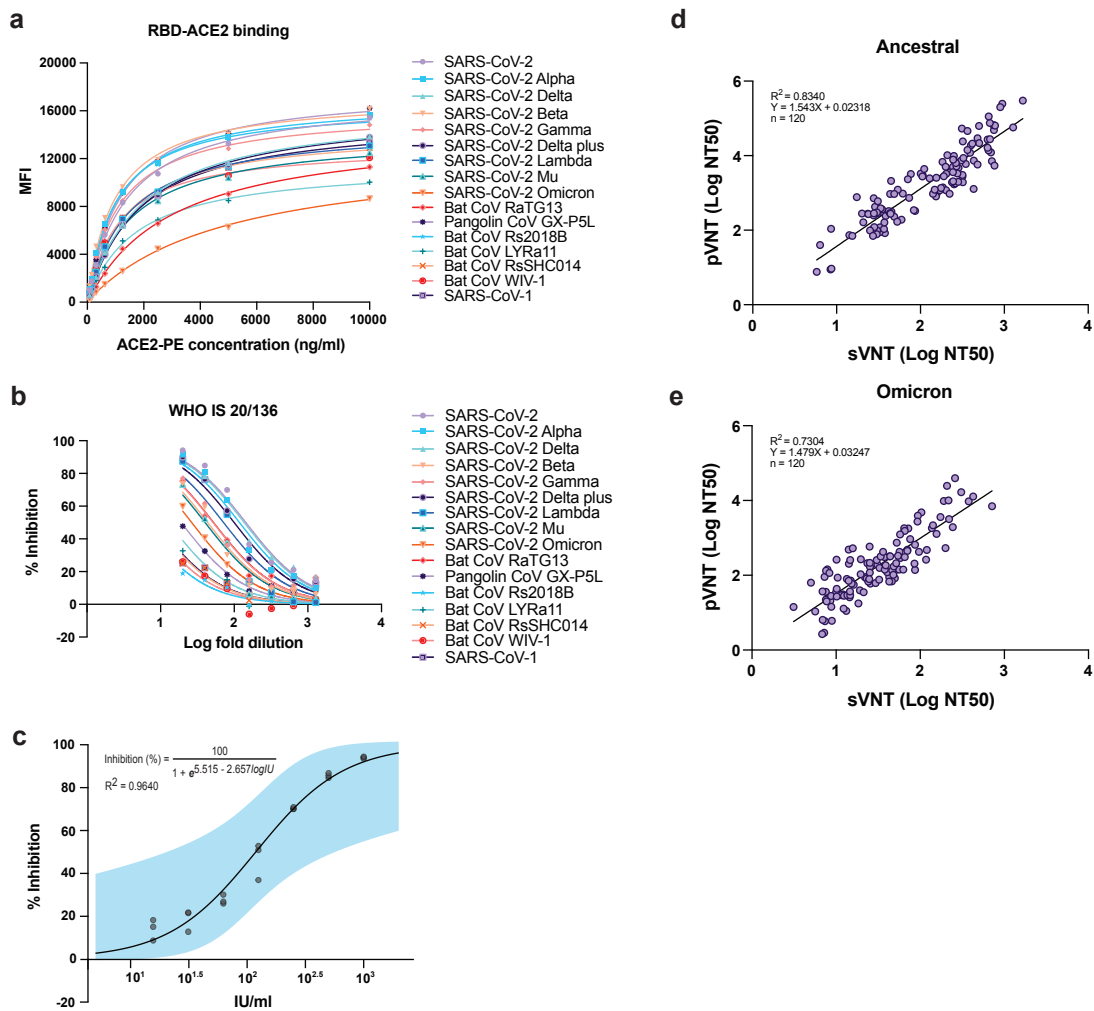

Supplement: Supplement 2 [file 5f20b64bac112a6ea491d94e.pdf]

**a**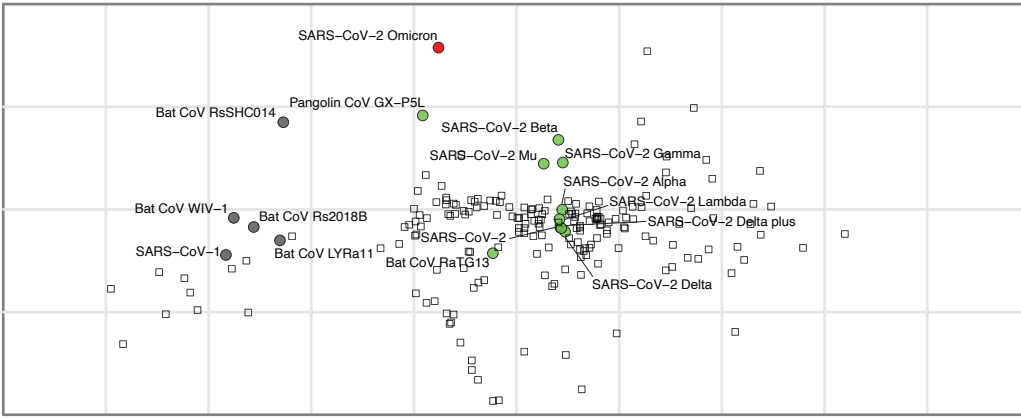**b**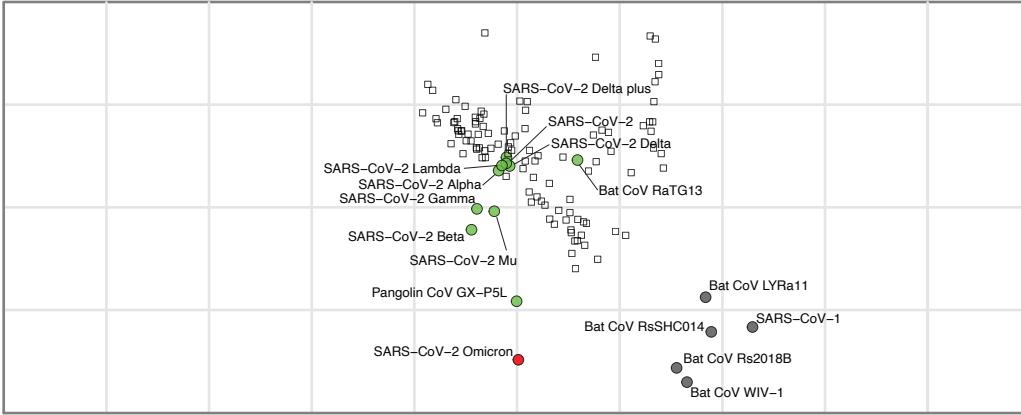**c**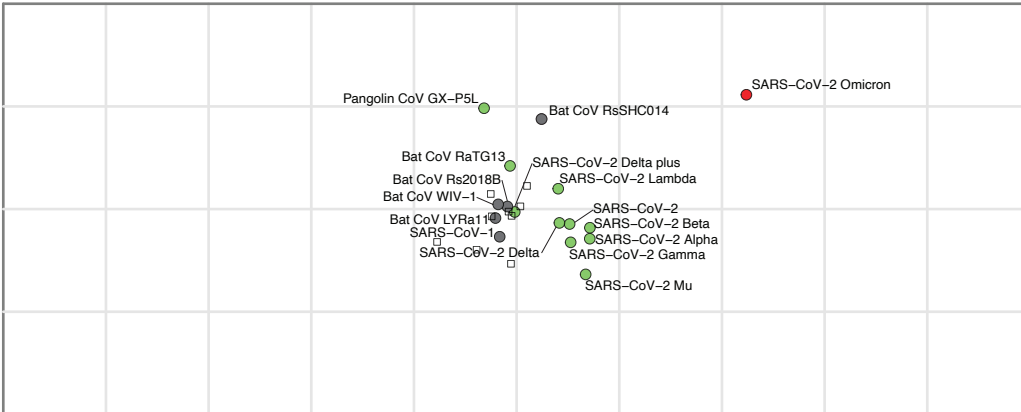

Supplement: Supplement 4 [file b3fd33f27dfee5aca1712d1c.pdf]
