# Supplementary material for: Differential escape of neutralizing antibodies by SARS-CoV-2 Omicron and pre-emergent sarbecoviruses
Source: Res Sq. 2022 Feb 23:rs.3.rs-1362541. Preprint. [Version 1] doi: 10.21203/rs.3.rs-1362541/v1 (PMC8887082; doi:10.21203/rs.3.rs-1362541/v1)
Supplement: Supplement 3 [file 874157dea35d1a312c3466f2.docx]

**Differential escape of neutralizing antibodies by SARS-CoV-2 Omicron and pre-emergent sarbecoviruses**

Chee Wah Tan^1,^*, Wan Ni Chia^1^, Feng Zhu^1^, Barnaby E Young^2,3,4^, Napaporn Chantasrisawad^5^, Shi-Hsia Hwa^6,7^, Aileen Ying-Yan Yeoh^1^, Beng Lee Lim^1^, Wee Chee Yap^1^, Surinder Kaur MS Pada^8^, Seow Yen Tan^2,10^ , Watsamon Jantarabenjakul^5,9^, Shiwei Chen^1^, Jinyan Zhang^1^, Yun Yan Mah^1^, Vivian Chih-Wei Chen^1^, Mark I-C Chen^2,3^, Supaporn Wacharapluesadee^5^, Alex Sigal^6,11,12^, Opass Putcharoen^5,8^, David Chien Lye^2,3,4,13^ and Lin-Fa Wang^1,^*

^1^Programme in Emerging Infectious Diseases, Duke-NUS Medical School, Singapore

^2^National Center of Infectious Diseases, Singapore

^3^Tan Tock Seng Hospital, Singapore

^4^Lee Kong Chian School of Medicine, Nanyang Technological University, Singapore.

^5^Thai Red Cross Emerging Infectious Diseases Clinical Center, King Chulalongkorn Memorial Hospital, Bangkok, Thailand

^6^Africa Health Research Institute, Durban, South Africa

^7^Division of Infection and Immunity, University College London, London, United Kingdom

^8^Ng Teng Fong General Hospital, Singapore

^9^Faculty of Medicine, Chulalongkorn University, Bangkok, Thailand

^10^Changi General Hospital, Singapore

^11^School of Laboratory Medicine and Medical Sciences, University of KwaZulu-Natal, Durban, South Africa

^12^Max Planck Institute for Infection Biology, Berlin, Germany

^13^Yong Loo Lin School of Medicine, National University of Singapore, Singapore

*Corresponding author: Lin-Fa Wang ([linfa.wang@duke-nus.edu.sg](mailto:linfa.wang@duke-nus.edu.sg)); Chee Wah Tan ([cheewah.tan@duke-nus.edu.sg](mailto:cheewah.tan@duke-nus.edu.sg)); Programme in Emerging Infectious Diseases,

Duke-NUS Medical School, Singapore.

**Table of contents**

**Supplementary Data Fig 1.**

**Supplementary Data Fig 2**

**Supplementary Data Table 1**

**Supplementary Data Table 2**

**
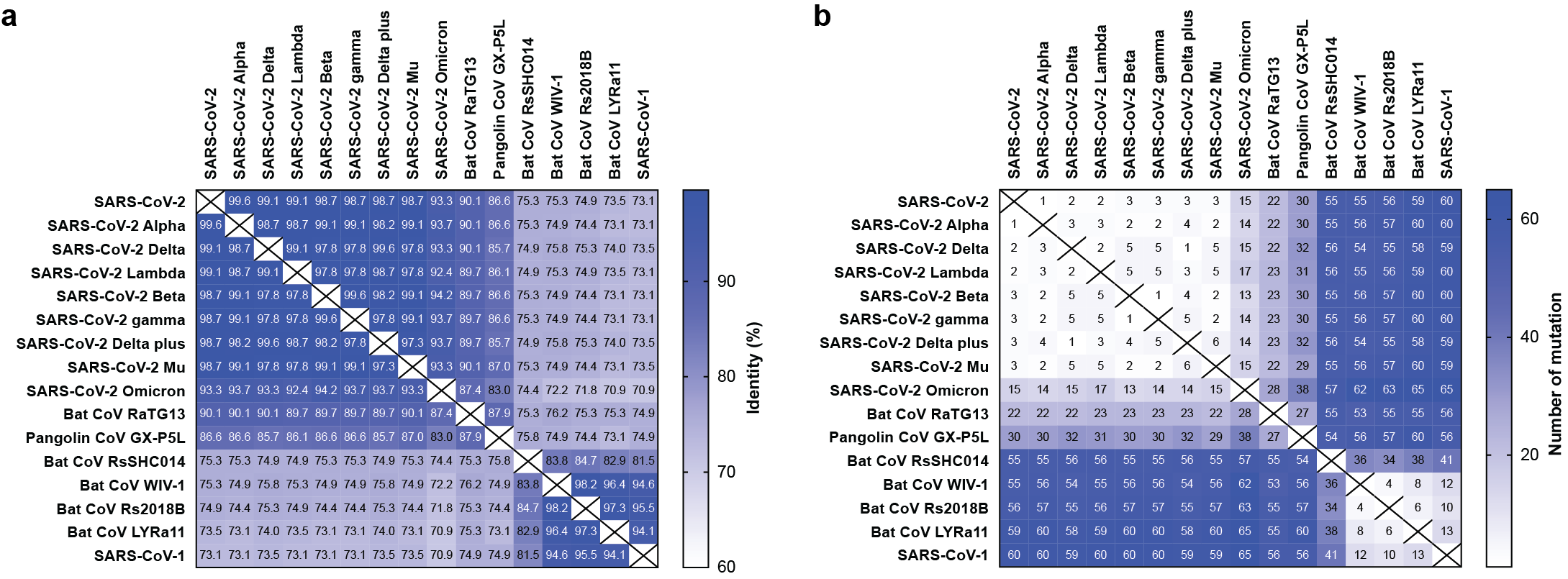
**

**Supplementary Data Fig 1. Amino acid difference between sarbecovirus RBDs.** Matrix of **(a)** amino acid identity (%) and **(b)** mutation numbers of 16 sarbecovirus RBDs used in multiplex sVNT. RBD sequences are aligned using Geneious alignment with Blosum62 model.

**
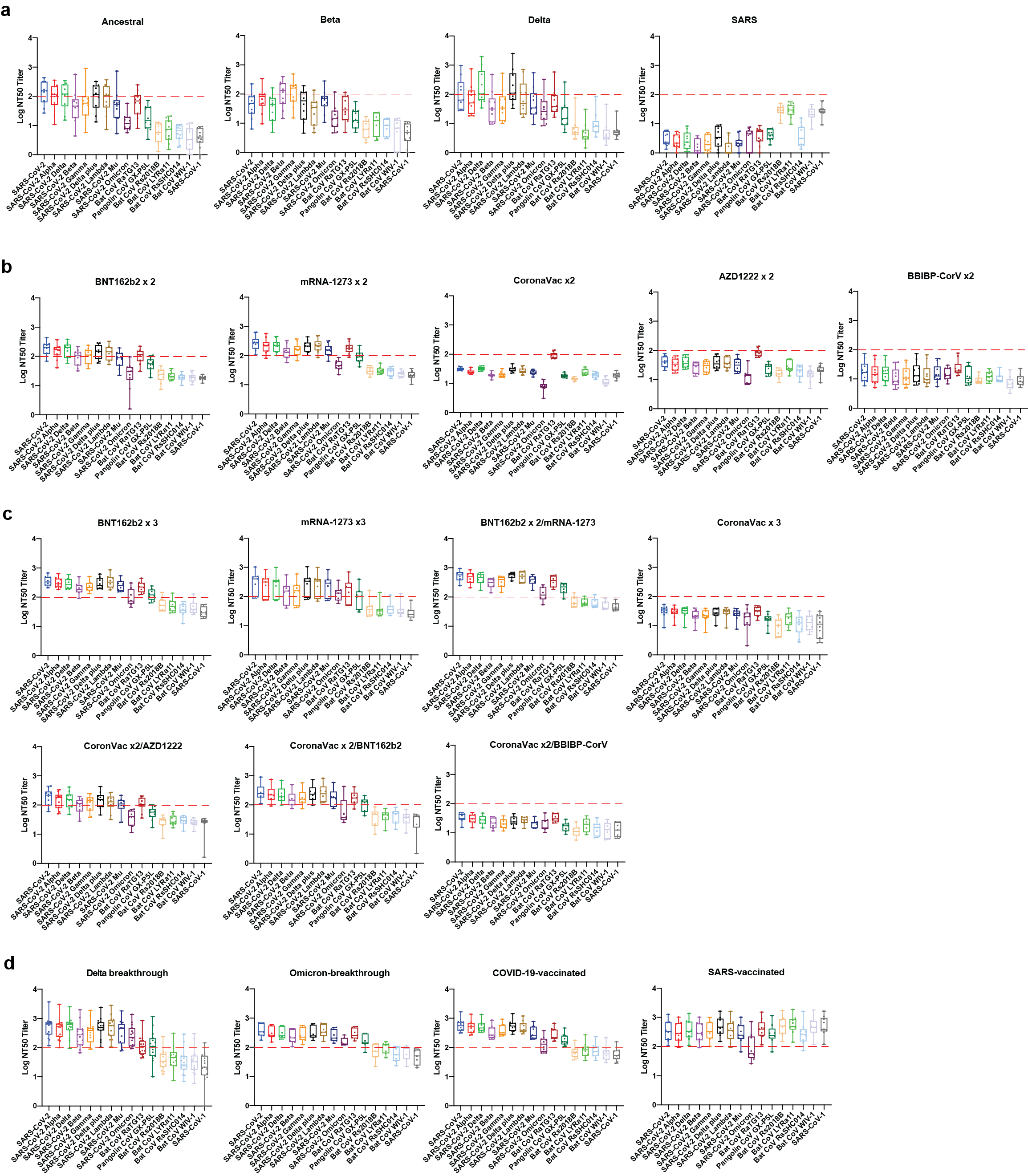
**

**Supplementary Data Fig 2. Neutralization titer 50% (NT50) of 20 serum panels determined using multiplex sVNT. (a)** Convalescent serum panels derived from SARS-CoV-2 ancestral, Beta, Delta and SARS-CoV-1 survivors. **(B)** Vaccinated serum panels derived from individuals received two doses of BNT162b2, mRNA-1273, CoronaVac, AZD1222 and BBIBP-CorV. **(C)** Boosted serum panels from individuals received homologous booster (BNT162b2 x3, mRNA-1273 x3, CoronaVac x3) and heterologous booster regime (BNT162b2 x2/mRNA-1273, CoronaVac x2/BNT162b2, CoronVac x2/AZD1222 and CoronaVac x 2/BBIBP-CorV). **(D)** Serum panels derived from individualds with hybrid immunity (SARS-vaccinated, COVID-19-vaccinated, Delta-breakthrough and Omicron-breakthrough).

**Supplementary Data Table 1. Serum panel and WHO IU/ml**

| **Group** | **Serum Panel** | **Number** | **Geometric mean inhibition (%)^a^** | **IU/ml** |
| --- | --- | --- | --- | --- |
| 1  (Convalescent) | Ancestral | 10 | 57.03 | 608 |
|  | Beta | 10 | 29.15 | 220 |
|  | Delta | 10 | 41.81 | 357 |
|  | SARS | 11 | 1.00 | 9 |
| 2  (Vaccinated) | BNT162b2 x 2 | 10 | 74.61 | 1211 |
|  | mRNA-1273 x 2 | 10 | 83.03 | 1884 |
|  | CoronVac x 2 | 10 | 24.27 | 177 |
|  | BBIBP-CorV x 2 | 10 | 14.87 | 105 |
|  | AZD1222 x 2 | 10 | 26.71 | 198 |
|  | BNT162b2 x 3 | 7 | 90.98 | 3526 |
|  | mRNA-1273 x 3 | 7 | 79.25 | 1520 |
|  | BNT162 x 2/mRNA-1273 | 6 | 94.18 | 5312 |
|  | CoronaVac x 3 | 10 | 23.64 | 172 |
|  | CoronaVac x 2/BBIBP-CorV | 7 | 24.60 | 180 |
|  | CoronaVac x 2/AZD1222 | 10 | 66.42 | 859 |
|  | CoronaVac x 2/BNT162b2 | 6 | 80.11 | 1592 |
| 3  (Hybrid) | Delta Breakthrough | 16 | 87.86 | 2644 |
|  | Omicron breakthrough | 7 | 88.98 | 2906 |
|  | COVID-19 vaccinated | 10 | 93.46 | 4768 |
|  | SARS vaccinated | 9 | 83.30 | 1914 |

^a^Geometric mean inhibition (%) of ancestral SARS-CoV-2 at 1:80 dilution.

**Supplementary Data Table 2. Serum panels and IRB approval.**

| **Group** | **Serum Panel** | **Number** | **IRB** |
| --- | --- | --- | --- |
| 1  (Convalescent) | Ancestral | 10 | Duke-NUS: LH-20-006EC  NHG DSRB 2012/00917 |
|  | Beta | 10 | University of KwaZulu–Natal BREC/00001275/2020 |
|  | Delta | 10 | Duke-NUS: LH-20-006EC  NHG DSRB 2012/00917  Faculty of Medicine, Chulalongkorn University, Bangkok, Thailand IRB no 291/63 |
|  | SARS | 11 | Duke-NUS: LH-20-006EC  NHG DSRB E 2020/00091 |
| 2  (Vaccinated) | BNT162b2 x 2 | 10 | NUS-IRB-2021-108 |
|  | mRNA-1273 x 2 | 10 | NUS-IRB-2021-108 |
|  | CoronVac x 2 | 10 | Faculty of Medicine, Chulalongkorn University, Bangkok, Thailand IRB no 170/64 |
|  | BBIBP-CorV x 2 | 10 | NUS-IRB-2021-108 |
|  | AZD1222 x 2 | 10 | Faculty of Medicine, Chulalongkorn University, Bangkok, Thailand IRB no 170/64 |
|  | BNT162b2 x 3 | 7 | NUS-IRB-2021-108 |
|  | mRNA-1273 x 3 | 7 | NUS-IRB-2021-108 |
|  | BNT162 x 2/mRNA-1273 | 6 | NUS-IRB-2021-108 |
|  | CoronaVac x 3 | 10 | NUS-IRB-2021-108 |
|  | CoronaVac x 2/BBIBP-CorV | 7 | NUS-IRB-2021-108 |
|  | CoronaVac x 2/AZD1222 | 10 | Faculty of Medicine, Chulalongkorn University, Bangkok, Thailand IRB no 170/64 |
|  | CoronaVac x 2/BNT162b2 | 6 | NUS-IRB-2021-108 |
| 3  (Hybrid) | Delta Breakthrough | 16 | Duke-NUS: LH-20-006EC  NHG DSRB 2012/00917 |
|  | Omicron breakthrough | 7 | Duke-NUS: LH-20-006EC  NHG DSRB 2012/00917 |
|  | COVID-19 vaccinated | 10 | Duke-NUS: NUS-IRB-2021-840 |
|  | SARS vaccinated | 9 | Duke-NUS: LH-20-006EC  Duke-NUS: NUS-IRB-2021-840 |
